# Field measurements of a massive *Porites* coral at Goolboodi (Orpheus Island), Great Barrier Reef

**DOI:** 10.1038/s41598-021-94818-w

**Published:** 2021-08-19

**Authors:** Adam Smith, Nathan Cook, Kailash Cook, Rachelle Brown, Richard Woodgett, John Veron, Vicki Saylor

**Affiliations:** 1 Reef Ecologic, 14 Cleveland Terrace, North Ward, Townsville, QLD 4810 Australia; 2 James Cook University, 1 James Cook Dr, Douglas, QLD 4811 Australia; 3 Reef Check Australia, 1/377 Montague Rd, West End, QLD 4101 Australia; 4 Coral Reef Research, P.O. Box 129, Millaa Millaa, QLD 4886 Australia; 5 Manbarra Aboriginal Corporation, 168 Pinnacle Drive, Condon, QLD 4815 Australia

**Keywords:** Ecology, Zoology, Environmental sciences, Ocean sciences

## Abstract

An exceptionally large coral *Porites* sp. has been identified and measured at Goolboodi (Orpheus Island), Great Barrier Reef (GBR). This coral was measured in March 2021 during citizen science research of coral reefs in the Palm Islands group. We conducted a literature review and consulted scientists to compare the size, age and health of the *Porites* with others in the GBR and internationally. This is the largest diameter *Porites* coral measured by scientists and the sixth highest coral measured in the GBR. The health of the Porites was assessed as very good with over 70% live coral cover and minor percentages of sponge, live coral rock and macroalgae. An estimated age of 421–438 years was calculated based on linear growth models. Manbarra Traditional Owners were consulted and suggested that the *Porites* be named Muga dhambi (big coral) to communicate traditional knowledge, language and culture to indigenous, tourists, scientists and students.

## Introduction

*Porites*, phylum Cnidaria, Order Scleractinia are a common genus of coral with a worldwide distribution and are represented by at least 16 species on the GBR^1,2^. Several species have massive growth forms and can grow to over 6 m in diameter and several metres high^3,4^. Massive *Porites* are some of the most important reef building corals and often constitute the major proportion of corals in nearshore locations^5–7^.

Done and Potts^8^ noted that large, massive *Porites* (up to 10 m) on the GBR are scarce and limited to localised aggregations in a specific zone of the reef front terrace (6–12 m depth), or on sheltered inshore reefs. Massive *Porites* display a variety of growth forms^3^. They are commonly referred to as bommies.

The state of a coral reef is most simply indicated by the variable “hard coral cover”^9^. Surprisingly few monitoring programs measure or even estimate the size of coral colonies^10^. Long-term declines in the colony size structure of coral populations along the GBR have been attributed to mass coral bleaching in 2016 and 2017^11^.

Potts et al.^6^ reported a very large, rounded *Porites* colony, 6.9 m in diameter, living on the GBR was at least 677 years old. This age has been queried as potentially inaccurate due to counting two growth bands per year as happens in equatorial latitudes^12^.

The aim of this note is to focus on the biology, health, resilience, social and cultural value of a significant coral bommie and to consider options for future protection, stewardship and communication.

## Materials and methods

**Figure 1:**
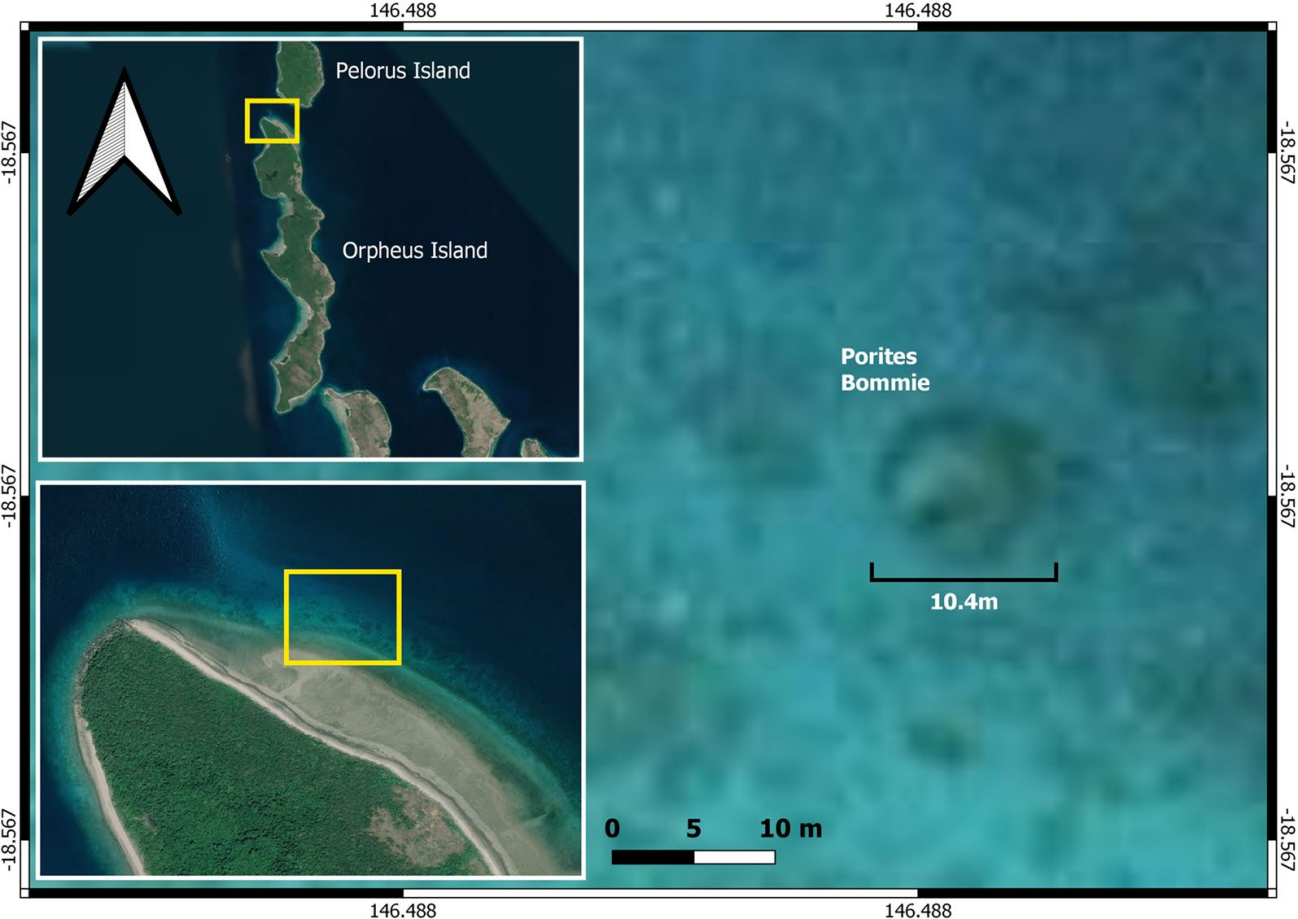
Map of the location of the *Porites* at Goolboodi (Orpheus) Island. Map created using QGIS 3.10 software using Google Maps plugin.

**Figure 2:**
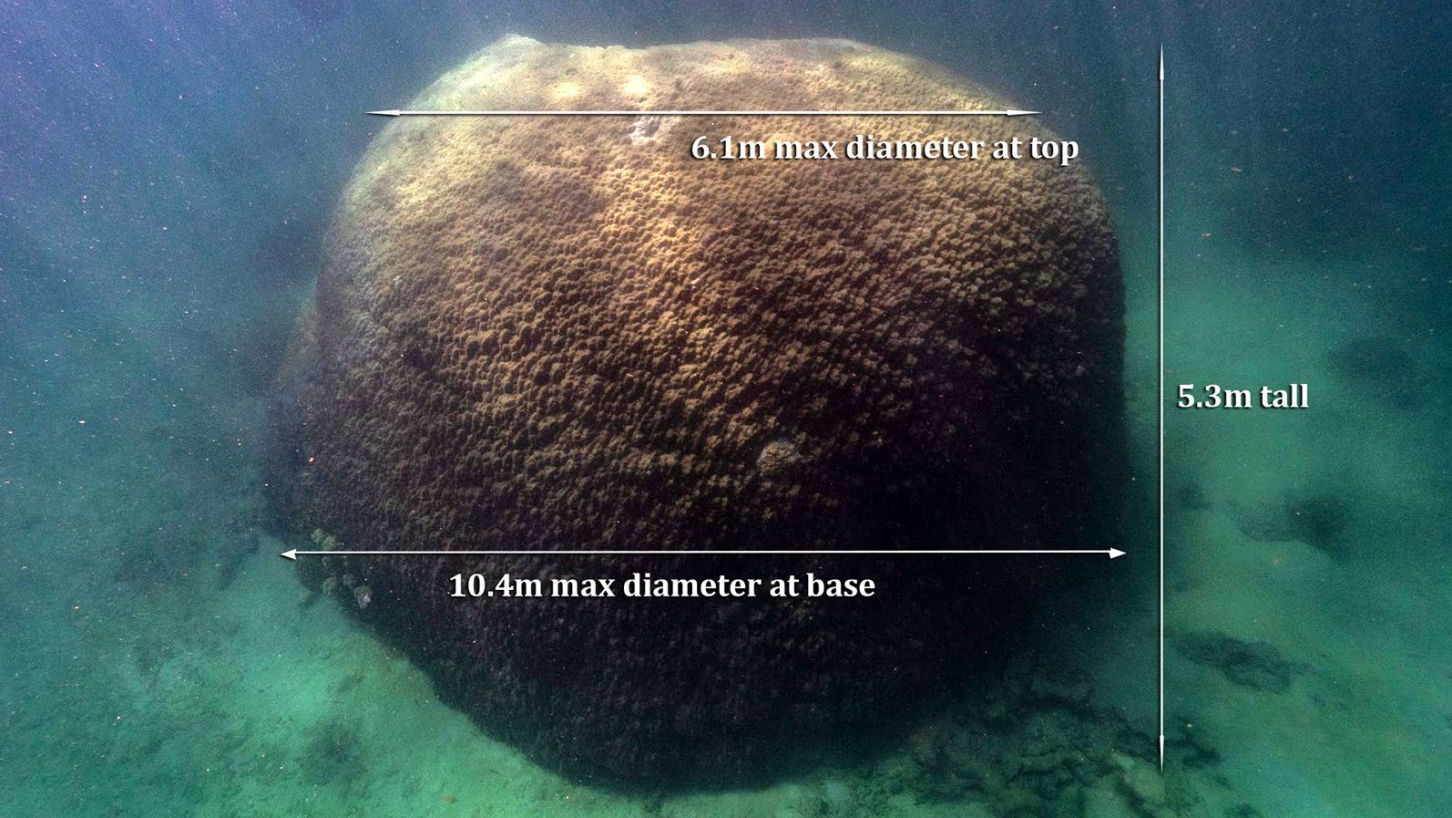
The front of the *Porites* sp. bommie noting the summary of measurements.

The *Porites* described in this field note was observed by snorkelers undertaking citizen science research at Goolboodi (Orpheus Island) (Fig. 1). It is located within a reef slope, on a sandy habitat in a maximum water depth of 7.4 m and the top of the bommie was 2.1 m below the surface (2.0 m tide). The coral was georeferenced, measured and photographed on 20 and 21 March 2021 (Fig. 2, Table 1). A 50 m transect tape was used to measure height (greatest colony distance perpendicular to the substrate), maximum diameter (planar diameter with greatest aerial projection onto the substrate) and width (diameter orthogonal to the maximum diameter measured at the center of the colony) (Table 1). We cross-checked the height and water depth with a Garmin GPSMAP 942 chart plotter and depth sounder.

**Table 1:** Location and measurements of *Porites* sp.

Date	Latitude	Longitude	Diameter (m)	Height (m)	Circumference (m)
20–21 March 2021	18.56701S	146.48843E	10.4	5.3	31

Three individual Reef Health Impact Surveys (RHIS)^13^ were undertaken to assess the benthic cover of the coral bommie. RHIS is a citizen science survey method developed by the Great Barrier Reef Marine Park Authority to enable rapid assessment of the benthic reef environment. It involves the surveyor randomly selecting a circle of reef with a 10-m diameter and estimating the benthos into six benthic categories—macroalgae, live coral, recently dead coral, live coral rock, rubble and sand. With a diameter of ten metres, the RHIS methodology was ideally suited to assess coral cover and benthos of the bommie. One limitation of RHIS is the exclusion of sponge in its record of reef benthos. As a portion of the bommie was occupied by an encrusting sponge, a separate category was added for the purpose of describing habitat.

## Results and discussion

The location, diameter, height and circumference of the coral were measured (Table 1, Fig. 2). The *Porites* was brown to cream in colour and hemispherical in shape (Fig. 2). It was identified as either *Porites lutea* (Hump or Pore coral) or *P. lobata* (Lobe coral)^14^.

**Figure 3:**
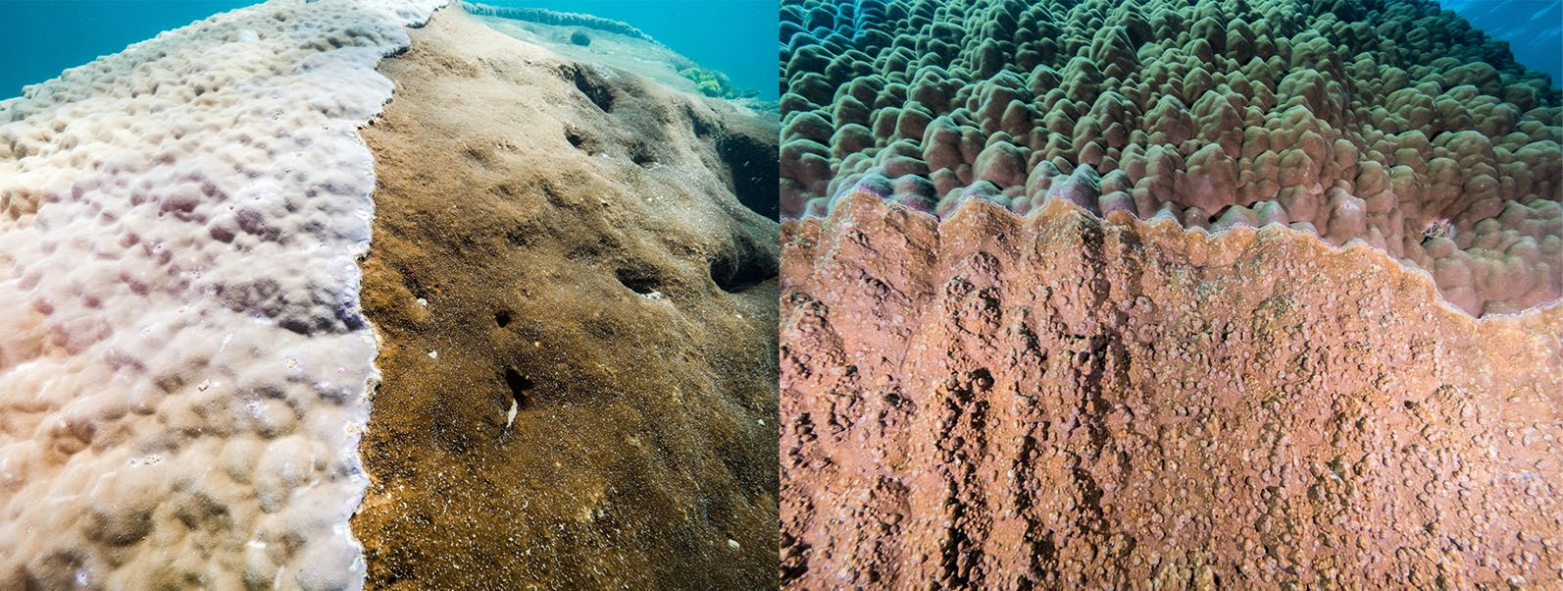
Detail of the sub-habitats and competitive interactions *Porites* sp. and boring sponge *Cliona viridis* (left) and live coral *Porites* sp. and *Montipora* sp. (right) along interspecific contact zones.

**Figure 4:**
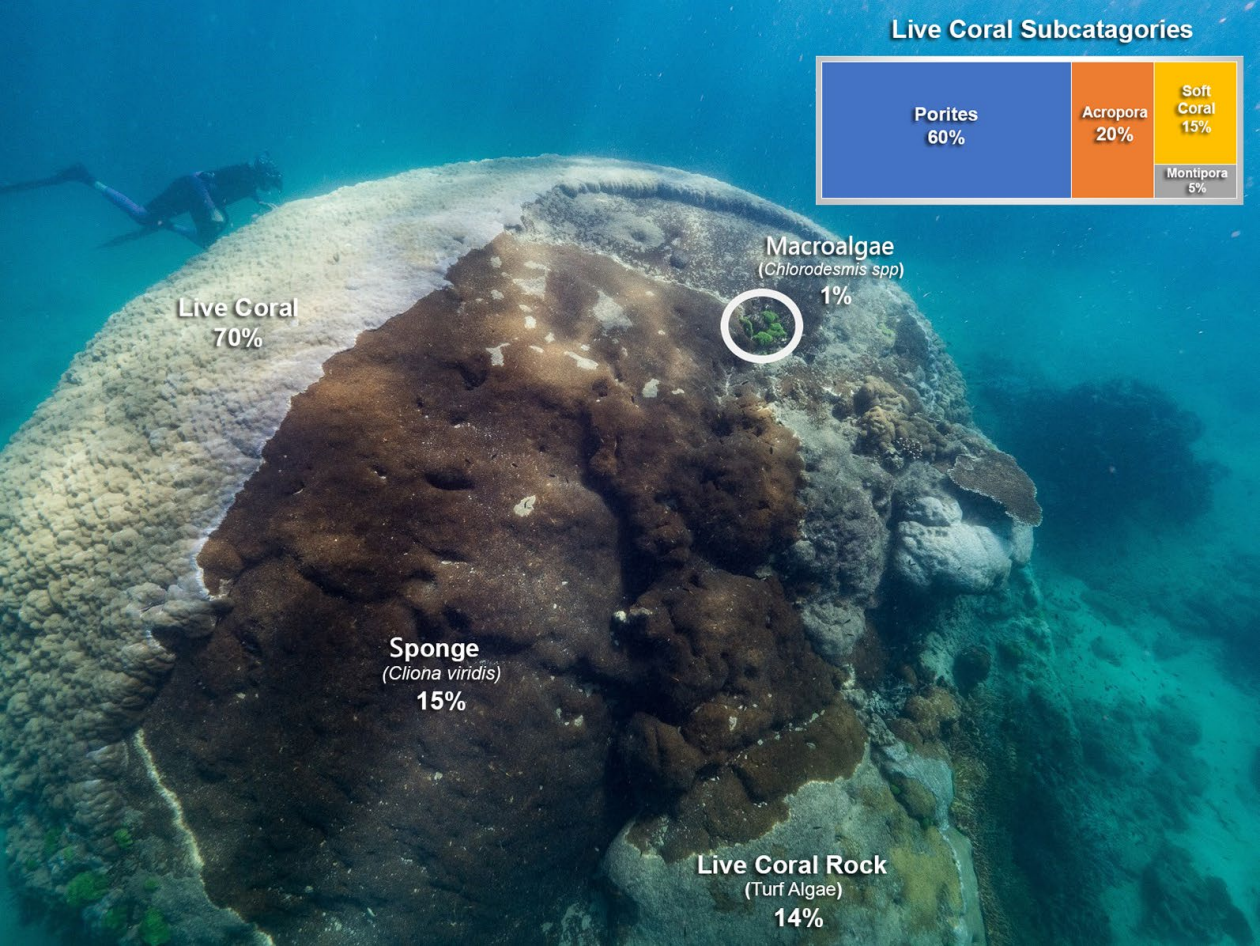
Detail of Reef Health Impact Survey (RHIS) of *Porites.*

The primary habitat on the *Porites* was live coral (70%), followed by sponge, live coral rock and a small amount of macroalgae (Table 2). No recently dead coral, coral rubble or sand was recorded (Table 2). We observed competition between the *Porites* and other species of coral and invertebrate including encrusting sponge, plating and branching *Acropora* spp., *Montipora, Chlorodesmis*, soft coral and zoanthids (Table 2, Figs. 3, 4).

**Table 2:** Reef Health Impact Survey (RHIS) of habitat and species categories on *Porites* sp.

	Macroalgae	Live Coral	Recently Dead Coral	Live Coral Rock	Coral Rubble	Sand	Sponge
Habitat	1%	70%	0%	14%	0%	0%	15%
Species	100% *Chlorodesmis*	60% *Porites* 20% *Acropora* 5% *Montipora* 15% Soft coral					*Cliona viridis*

The boring sponge, *Cliona viridis,* is abundant on the Great Barrier Reef^15^. It is a common bioeroding species advancing laterally at around 1 cm and to a depth of 1.2 cm per annum^15^. Abundance of *Cliona viridis* is often correlated to substrate availability and water energy with the greatest abundance often on the windward side of bommies^15^. This correlates to our observations as the large proportion of the substrate estimated to cover the bommie (15%) was on the windward side. The sponge’s advances will likely continue to compromise the colony size and health.

We recorded marine debris at the base of the *Porites*. The debris was 2–3 m of rope that appeared to have been wrapped around the base of an adjacent coral. Adjacent to the bommie were three concrete blocks.

How big is the *Porites* coral at Goolboodi compared to other big corals in the GBR, and the world? Potts et al.^6^ reported a very large, rounded *Porites* colony, 6.9 m in diameter which is 3.1 m smaller than this study. Lough et al.^16^ reported coral cores from colonies between 1.6–8.0 m in height with the largest corals of 6.0 m at Havannah, North Molle and Masthead Islands, 7.5 m at Abraham Reef and 8.0 m at Sanctuary Reef. Recognising the limitations of published data, the *Porites* coral at Goolboodi is the largest diameter coral that has been measured, and the 6th tallest in the GBR. It is unknown if the other corals are still alive or dead.

Other comparatively large massive *Porites* have previously been located throughout the Pacific. These have included multiple bommies measuring more than 10 m^4^ and one exceptionally large colony observed measuring 17 m × 12 m in American Samoa^17^. Additionally, large *Porites* sp. bommies have been observed at Green Island, 30 km east of Taiwan^18^ as well as an 11 m diameter *Porites australiensis* at Sesoko Island, Okinawa, Japan^19^.

How old is this massive *Porites*? In discussions with the Australian Institute of Marine Science (AIMS), there is a robust, linear relationship (> 80% variance explained) between *Porites* average linear extension rate and average annual sea surface temperature (SST)^20,21^ that provides an estimate of colony age from its height. Using average annual SST at 18.5S, 146.5E of 26.12C (from HadiSST data set), the estimated linear extension rate is determined by (2.97 × 26.12) − 65.46 = 1.21 cm/year. Given the colony height of 5.1–5.3 m, this gives an estimated age of 421–438 years. This is well before European exploration and settlement of Australia. AIMS has investigated over 328 colonies of massive *Porites* corals from 69 reefs along the GBR and has aged them as being from 10–436 years^21^. AIMS has not investigated this coral (pers. comm Neal Cantin). Based on limitations of published data, the *Porites* coral at Goolboodi is one of the oldest corals on the GBR.

Why is the *Porites* partially dead on top and living on the side? The proportion of live coral tissue on a colony reflects the cumulative, integrated effect of both beneficial and adverse environmental factors. Substantial portions of coral tissue can die from exposure to sun at low tides or warm water without lethal consequences to the colony as a whole^10^. Partial mortality of large bommies provides available real estate for opportunistic, fast growing sessile organisms. In this instance, multiple species of tabulate and branching *Acropora* sp., encrusting *Montipora* sp. and encrusting sponges are among the benthic organisms to have colonised 30% of the coral bommies’ surface area. Intraspecific competition is also evident from the skeletal barriers created along contact zones^22^ (Fig. 3). There was no observation of disease or coral bleaching.

The *Porites* is located in a relatively remote, rarely visited and highly protected Marine National Park (green) zone. Its location had not been previously reported and there is no existing database for significant corals in Australia or globally. Cataloguing the location of massive and long-lived corals can have multiple benefits. Scientific benefits include geochemical and isotopic analyses in coral skeletal cores which can help understand century-scale changes in oceanographic events and can be used to verify climate models. Social and economic benefits can include diving tourism, citizen science^23^ culture and stewardship. Perhaps the Significant Trees Register, which was designed by the National Trust^24^ to protect and celebrate Australia’s heritage could be considered as a model. There are risks of cataloguing the location of massive corals. It could be damaged by direct and indirect human uses including anchoring, research and pollution.

Indigenous languages are an integral part of Indigenous culture, spirituality, and connection to country. We consulted Manbarra Traditional Owners about protocol and an appropriate cultural name for the *Porites* and they considered: Big (Muga), Home (Wanga), Coral reef (Muugar), Coral (Dhambi), Old (Anki, Gurgu), Old man (Gulula) and Old person (Gurgurbu)^25^. The recommendation by Manbarra Traditional Owners is that the *Porites* is named as Muga dhambi (Big coral). The feedback from the process of consultation was very positive with acknowledgement of the respect that the scientists have demonstrated to acknowledge Traditional Owners in this way.

The large *Porites* coral at Goolboodi (Orpheus) Island is unusually rare and resilient. It has survived coral bleaching, invasive species, cyclones, severely low tides and human activities for almost 500 years. In an attempt to contextualise the resilience of these individual *Porites* we have reviewed major historic disturbances such as coral bleaching which has occurred since at least 1575 and potentially 99 bleaching events in the GBR over the past 400 plus years^26^. Other indicators such as high-density ‘stress bands’ were recorded from 1877 and are significantly more frequent in the late twentieth and early twenty-first centuries in accordance with rising temperatures from anthropogenic global warming^27^. In addition there have been an average of 1–2 tropical cyclones per decade (40–80 in total) that have potentially impacted the coral adjacent to Goolboodi Island^28,29^; 46 tropical cyclones impacted the area between Ingham and Townsville from 1858 to 2008^30^. The cumulative impact of almost 100 bleaching events and up to 80 major cyclones over a period of four centuries, plus declining nearshore water quality contextualise the high resilience of this *Porites* coral. Looking to the future there is real concern for corals in the GBR due to many impacts including climate change, declining water quality, overfishing and coastal development^31,32^. This field note provides important geospatial, environmental, and cultural information of a rare coral that can be monitored, appreciated, potentially restored and hopefully inspire future generations to care more for our reefs and culture.
